# A miniature robotic steerable endoscope for maxillary sinus surgery called PliENT

**DOI:** 10.1038/s41598-022-05969-3

**Published:** 2022-02-10

**Authors:** Julie Legrand, Mouloud Ourak, Laura Van Gerven, Vincent Vander Poorten, Emmanuel Vander Poorten

**Affiliations:** 1 Robotics, Automation and Mechatronics (RAM) Group, KU Leuven, Leuven, Belgium; 2 Otorhinolaryngology, Head Neck Surgery, University Hospitals Leuven, Leuven, Belgium; 3 Department of Microbiology, Immunology and Transplantation, Allergy and Clinical Immunology Research Unit, KU Leuven, Leuven, Belgium; 4 Department of Neurosciences, Experimental Otorhinolaryngology, Rhinology Research, KU Leuven, Leuven, Belgium; 5 Department of Oncology, Section Head and Neck Oncology, KU Leuven, Leuven, Belgium

**Keywords:** Biomedical engineering, Surgery, Therapeutic endoscopy

## Abstract

In endoscopic maxillary sinus surgery, the maxillary sinus is accessed through the nasal cavity which constitutes a narrow and tortuous pathway. However, surgeons still use rigid endoscopes and rigid, straight or pre-bent instruments for this procedure. Resection of the uncinate process and creation of a medial antrostomy is warranted to access the pathology inside the maxillary sinus and depending on the location of the pathology (lateral, inferior or anterior wall), additional resection of healthy tissue and/or functional structures like the lacrimal duct and/or inferior turbinate is necessary to gain optimal access. In order to avoid this additional resection, a functional single-handed, steerable endoscope for endoscopic maxillary sinus surgery has been designed and built. This endoscope is, to our knowledge, the most slender active steerable endoscope ever reported for maxillary sinus surgery. The performance of the endoscope was validated by two surgeons on a cadaver. An increased field of view was found in comparison to currently used endoscopes. As a direct consequence, a reduced need for resection of healthy tissue was confirmed.

## Introduction

**Figure 1:**
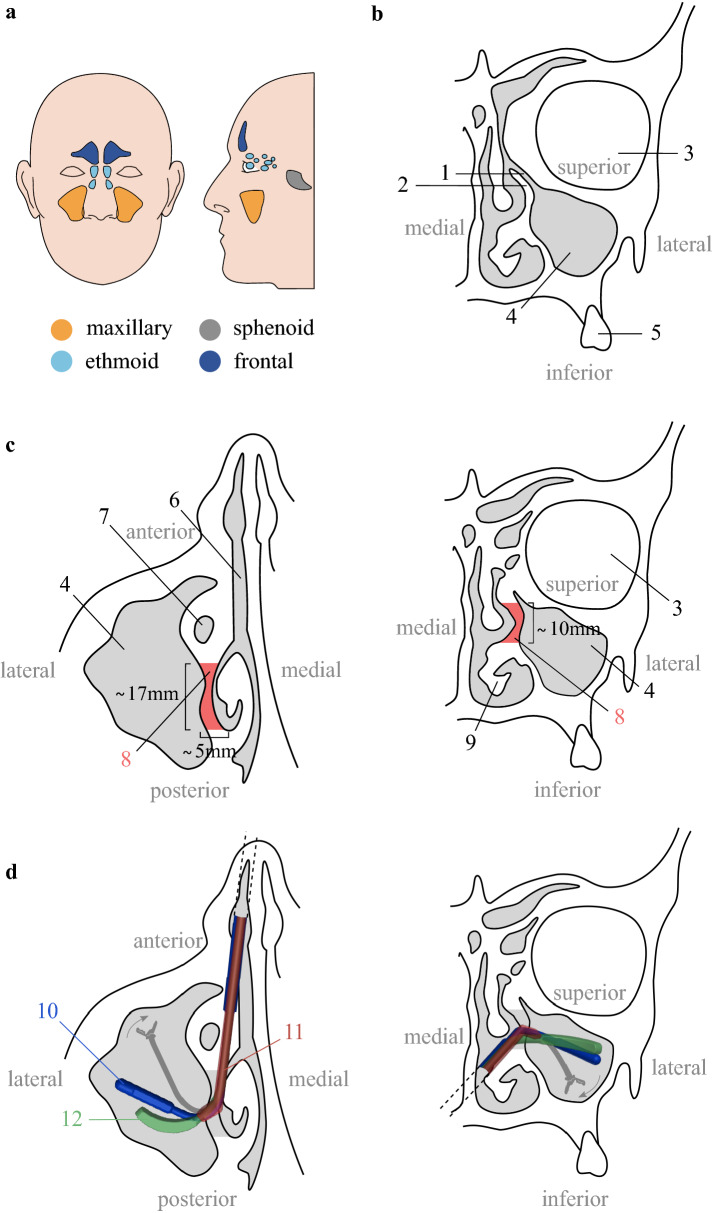
Maxillary sinus anatomy. (**a**),Location of the paranasal sinuses (Adapted from^2^); (**b**) Maxillary sinus and nasal cavity anatomy in the coronal plane; (**c**) Maxillary sinus and nasal cavity anatomy with medial antrostomy dimensions and procedure in the axial (left) and coronal plane (right): resection of the tissues in red; (**d**) Insertion of some conventional instruments for sinus surgery into the maxillary sinus: transversal (left) and coronal (right) views: a 3.2 mm diameter forceps $45^{\circ }$ upturned (11), a 2.5 mm diameter Heuwieser grasping forceps (12) and a 4 mm diameter double spoon forceps of $110^{\circ }$ (10), and finally, insertion of an envisioned dexterous flexible instrument (gray). The gray surfaces represent cavities. The white areas are bony structures. 1. maxillary ostium; 2. uncinate process; 3. orbit; 4. maxillary sinus; 5. molar dentition; 6. nasal cavity; 7. lacrimal canal; 8. antrostomy; 9. inferior turbinate.

The maxillary sinuses are the largest of the paranasal sinuses. They have a pyramid shape and are situated on either side of the nostrils (Fig. 1a). Each maxillary sinus is composed out of six bony walls: the anterior, lateral, posterior, superior, medial, and inferior walls (Fig. 1c). The anterior and lateral walls of the maxillary sinus are formed by the facial surface of the maxilla, whereas the posterior wall is formed by the infratemporal surface. The superior wall is formed by the fragile, triangular orbital floor. The medial wall of the maxillary sinus is rectangular in shape. It separates the sinus from the nasal cavity. The medial wall also contains a small opening (2–4 mm diameter), situated behind the uncinate process. This opening is referred to as the maxillary ostium or the maxillary hiatus. The maxillary ostium forms the drainage channel of the maxillary sinus (Fig. 1b). Finally, the floor or inferior wall of the sinus is separated from the molar dentition by a thin layer of bone (Fig. 1b)^1^.

Different pathologies can be found on the maxillary sinus walls: inverted papillomas, fungus balls and carcinomas. Those conditions require surgery. Endoscopic maxillary sinus surgery has become one of the main surgical procedures for maxillary sinus diseases today^3^. The procedure consists in inserting a rigid endoscope and instruments through the nasal cavity to reach the maxillary sinus and treat the pathological tissue(s). To do so, a medial meatal antrostomy has to be created^4,5^. This means that the surgeon creates a large opening, including the natural ostium of the maxillary sinus. More specifically, the surgeon removes the uncinate process (Fig. 1b) and the surrounding tissues such that a window is created from the posterior border of the lacrimal canal to the posterior wall of the maxillary sinus (window length approximates 17 mm) and from the superior border of the inferior turbinate to the orbit floor (window height approximates 10 mm) (Fig. 1c). One of the purpose of this window is to restore proper drainage and ventilation of the sinus^6^. This medial antrostomy also allows the surgeon to properly inspect the maxillary sinus cavity pre- and postoperatively^5^. The instruments used for maxillary sinus surgery depend on the pathology and its location with respect to the medial antrostomy. Currently, a broad range of rigid, straight or fixed-angled (up to $120^{\circ }$) instruments are used during maxillary sinus surgery^7^. The diameter of those instruments varies between 2 and 3mm. For visualization, straight, rigid 4mm external diameter endoscopes with a fixed angle of view of 0, 30, 45 or $70 ^{\circ }$ are used^7,8^. From the author’s experience, 0 and $30^{\circ }$ scopes are most commonly used for surgery whereas $70^{\circ }$ scopes are only used for inspection and follow-up because of the challenging eye-hand coordination associated with those endoscopes.

Unfortunately, with conventional rigid endoscopes and instruments, a medial antrostomy may not suffice to visualize and reach all kinds of pathologies in the maxillary sinus cavity. This is especially the case if the disease is situated on the anterior, antero-lateral or inferior wall (Fig. 1d). In these specific cases, more invasive approaches that widen the access are needed. For example, the nasolacrimal canal and the inferior turbinate may be resected^9^, or a prelacrimal approach may need to be used^10^. Alternatively, an aperture in the canine fossa may be created (Caldwell–Luc procedure)^11,12^. These extra openings could lead to extra perioperative and postoperative complications^13^. Our personal experience is that more invasive approaches are needed in about 20% of isolated maxillary lesions (data unpublished).

Tichenor et al. summarized the possible advantages of flexible over rigid endoscopes to examine the maxillary sinus post-surgery^14^. They emphasized the ease of use of a flexible endoscope and the capability to maneuver into narrow recesses. However, the flexible endoscopes discussed by Tichenor et al. are passive (i.e. not robotically steerable) and therefore require two hands for operation, which requires the assistance of an extra surgeon during the procedure^14^. Schneider et al. also raised the unmet need for small flexible robotic instruments and endoscopes for sinus surgery^15^. In clinical practice, surgeons still resort to rigid instruments up to now. This work is therefore dedicated to the design and validation of a robotic, single handed, steerable endoscope with flexible tip for maxillary sinus surgery. Figure 1d shows a conceptual sketch of such an instrument that is inserted into the maxillary sinus. From the figure, it is clear that such a flexible instrument could potentially reach the anterior, antero-lateral and inferior walls.

In order to enhance currently used instruments, KARL STORZ launched the EndoCAMeleon (KARL STORZ, Tuttlingen, Germany)^16^. The EndoCAMeleon is a rigid endoscope with adjustable lens angle that offers an adjustable field of view from 0 to 120°, by rotating a control wheel situated on the instrument handle. A disadvantage of this device is that the surgeon would need to use both hands to change the endoscope’s field of view^17^. This means that a second surgeon would be needed if additional instruments ought to be manipulated. Moreover, the image of the EndoCAMeleon has been found to show a certain amount of distortion due to the lens inclination^18^.

Also in the scientific literature, flexible, steerable instruments for endoscopic maxillary sinus surgery have made their appearance. Table 1 summarizes these instruments. The table lists forseen per instrument, the respective diameter, performance characteristics such as the degree(s) of freedom (DOF), the maximum bending angle (BA) and the minimum bending radius (BR), as well as the suggested way of operation. Note that if the DOF is limited to a single direction, this is mentioned specifically as “1dir” in the one before last column.

The instruments of Yoon, Rosen and Hong et al. show large bending angles, which is promising for use in the maxillary sinus^19^. However, their diameters are relatively large (≥ 4 mm), which would require unnecessarily resection of healthy tissue to pass the nasal cavity^19^. Larger diameters also hamper simultaneous use of multiple tools inside the nasal cavity. Moreover, those instruments do not compensate for their large external diameter by foreseeing space to insert more than one tool inside the instrument shaft. Rosen et al. foresee multiple tools, namely: two scanning fibers, one forceps, one pair of scissors and one irrigation channel. But this functionality comes at the cost of an excessively large instrument diameter (8 mm). Yoon, Rosen and Hang intend to steer their instruments through teleoperation. Teleoperation has been proven useful in various surgical interventions^20^, but it would imply in this setting that operation happens at a greater distance separated from the patient and that the surgeon would lose force feedback, which can result in excessive force applied on the tissue causing tissue damage^21^. In contrast, the current practice allows the surgeons to enjoy full control and direct haptic feedback, which allows them to avoid the application of overly large stresses on the maxillary sinus. Especially in maxillary sinus surgery, where the optical nerve is situated in the operative field, uncontrolled force application could cause serious ophthalmic complications^22^. The importance of full control in direct contact has been repeatedly highlighted in prior literature^23,24^.

The concentric tube continuum robot of Webster et al. has a small external diameter. However, the bending radius of this instrument is overly large due to the inherent mechanics and material properties of concentric tube robots^25^. This prevents a proper access to the maxillary sinus^19^. Moreover, teleoperation is the preferred way to steer concentric tubes, as these devices are not easily controllable by hand^26^.

The Peregrine drivable ENTScope of 3NT Medical Ltd is relatively small (2.3 mm diameter), shows a large bending angle ($125 ^{\circ }$) and small bending radius (3 mm). However, this endoscope does not foresee a light source. The system is manually operated by the surgeon but actuation is passive. In contrast to active systems, surgeons often need both hands to operate the instrument: the first hand to hold the instrument in position and the second to manipulate or maintain the steering lever^27^. Even though this instrument shows good potential for endoscopic maxillary sinus surgery, Van Zele et al. report that the instrument does not allow ergonomic single-handed operation^28^, preventing the use of extra tools along with the endoscope by one surgeon only.

Also, the steerable forceps of Endius Inc. and Smith & Nephew feature small 3mm diameter and large bending angles (180° and 240°, respectively). But, also here, actuation is passive, forcing two-handed use.

From this state-of-the-art analysis of instruments for maxillary sinus surgery and their current limitations, we conclude that, there are to the best of our knowledge, up to now, no small diameter, large bending angle, user friendly, single-handed for maxillary sinus surgery. The main contribution of this work is to introduce the design and clinical validation of *PliENT*, an innovative, slender, steerable endoscope. The endoscope can be used for maxillary sinus surgery through a classical medial antrostomy. The small size and the flexibility of the instrument allows to limit the removal of additional functional structures like the lacrimal duct or the inferior turbinates. Moreover, its flexibility allows a wide range of angles of view without having to remove the endoscope from the operative field.

**Table 1:** State of the art of flexible, steerable instruments for endoscopic maxillary sinus surgery.

Author	Illustration	Tools	Diameter	Performance characteristics	Control
Yoon et al.^29^		Camera	4 mm	1 DOF; 180° BA; 9.5 mm BR	Teleoperation
Yoon et al.^29^		Gripper	5 mm	2 DOF; 180° BA; 9.5 mm BR	Teleoperation
Rosen et al.^30^		2 scanning fiber, forceps, scissors, irrigation channel	8 mm	3 DOF; 180° BA	Teleoperation
Hong et al.^31^		Gripper	4 mm	1 DOF; 270° BA; 10 mm BR	Teleoperation
Webster et al.^32^		–	0.8–2.4 mm	6 DOF	Teleoperation
3NT Medical Ltd^28^		Irrigation channel, camera	2.3 mm	1 DOF (1.dir); 125° BA; 3 mm BR	Manual (2 hands)
Endius Inc.^33^		Forceps	3 mm	1 DOF (1.dir); 180° BA	Manual (2 hands)
Smith & Nephew^34^		Forceps	3 mm	1 DOF; 240° BA	Manual (2 hands)

For the performance characteristics, the number of degree of freedom (DOF), the bending angle (BA) and the bending radius (BR) of each instrument is indicated when mentioned by the authors.

## Results and discussion

### Single-handed, flexible, steerable endoscope

**Figure 2:**
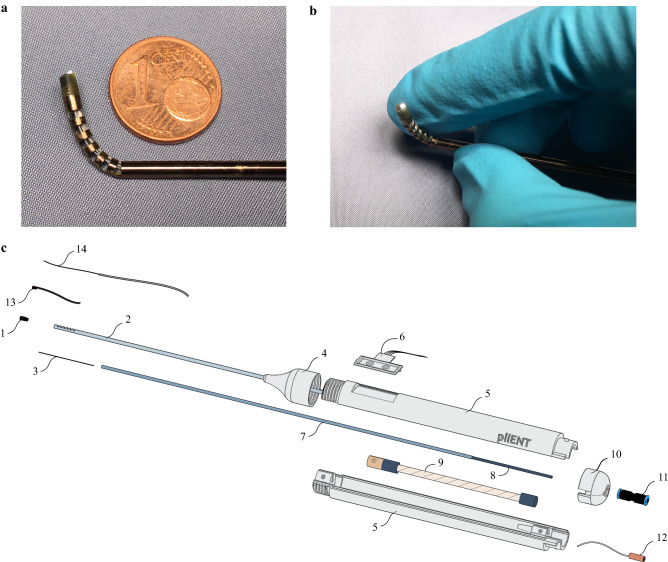
Flexible endoscope for maxillary sinus inspection. (**a**), view of the bending capabilities of the 2.3 mm diameter endoscope; (**b**), detail of the endoscope distal tip with camera and illumination; (**c**), overview of the different components of the single-handed, flexible, steerable endoscope for maxillary sinus surgery. 1. tip; 2. NiTi shaft; 3. cable ; 4. screw-on cap; 5. two-parts-handle; 6. button interface; 7. mobile outer tube of the concentric muscle; 8. fixed inner tube of the concentric muscle; 9. McKibben muscle; 10. plug-on cap; 11. pressure source connector; 12. pressure source tube; 13. chip-on-tip camera; 14. light fiber.

The PliENT endoscope is 370 mm long. Its shaft has a diameter of 2.3 mm and the handle, a diameter of 22 mm. The PliENT endoscope is, as far as the author is aware of, the most slender active flexible endoscope ever made for maxillary sinus surgery (Fig. 2). It’s assembly and control is detailed in the method section. The Supplementary Video V1 online shows the actuation of the PliENT endoscope.

### Maxillary sinus inspection

#### Experimental set-up

**Figure 3:**
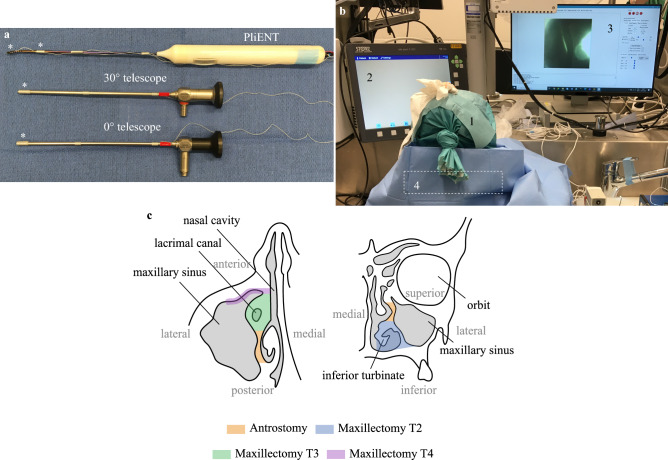
Experimental set-up and protocol for maxillary sinus inspection. (**a**) instruments used for the inspection of the cadaver’s maxillary sinus. Single-handed, flexible, steerable endoscope *PliENT* (top); Hopkins II forward-oblique rigid endoscope 30° (Karl Storz, Tuttlingen, Germany) (middle); Hopkins II straight forward rigid endoscope 0° (Karl Storz, Tuttlingen, Germany) (bottom). The locations of the 6 DOF electromagnetic sensors are indicated with the star symbol (*); (**b**) cadaver experiments set up consisting out of: 1. cadaver’s head; 2. Tele pack X LED for endoscopic view of the rigid endoscopes; 3. NanEye viewer for endoscopic view of the PliENT endoscope; 4. Aurora electromagnetic tracking system box; (**c**) maxillary sinus and nasal cavity anatomy in the transverse (left) and coronal (right) plane. The different enlargement procedures are colored as follows: orange = antrostomy; blue = maxillectomy type 2; green = maxillectomy type 3; purple = maxillectomy type 4.

In order to prove the added value of the PliENT endoscope in comparison to the currently used endoscopes, experiments were conducted on a cadaver specimen (male, 91 years old). Indeed, experiments on a realistic 3D printed phantom was no option. Indeed, the specific texture and colors of all the tissues present in the maxillary sinus (bone, cartilage, mucosa, blood vessels, nerves, ...) represent important anatomical “marker points” that allow the surgeons to navigate inside the sinus. To our knowledge, there is no realistic phantom available that take those tissue properties into account. Therefore, experiments on phantoms could lead to erroneous results as the surgeons could make mistakes in identifying the maxillary sinus walls due to the poor rendering of the maxillary sinus tissues. During the experiments, three instruments were tested, namely: a Hopkins II straight forward endoscope 0° (KARL STORZ, Tuttlingen, Germany), a Hopkins II forward-oblique endoscope 30° (KARL STORZ, Tuttlingen, Germany)^8^, and the PliENT endoscope (Fig. 3a). According to the authors’ experience, 0 and 30° endoscopes are the most commonly used endoscopes during endoscopic sinus surgery. The 70° scope is rather used for inspection and follow-up because of its challenging eye-hand coordination. During the entire duration of the experiments, the endoscopic views of each instrument were recorded. The tip position of each instrument was also recorded by using an electromagnetic tracking system (Aurora, NDI, Ontario, Canada) set up to track a 6 DOF sensor that was fixed to the tip of each instrument. For the PliENT endoscope, a second 6 DOF sensor was fixed on the straight, rigid part of the instrument shaft. This sensor was used as a reference. From the pair of sensors, it was possible to derive the instrument bending angle (Fig. 3a).

The cadaver’s head was placed on a plastic ring which allowed to maintain the head fixed during the experiments. The head and the ring were placed above the field generator of the electromagnetic tracking system (Fig. 3b). The Tele pack X LED (Karl Storz, Tuttlingen, Germany) was used to display the endoscopic view from the rigid endoscopes. A second screen was used to display the endoscopic view of the PliENT endoscope. The image from the PliENT enoscope was visualized by using the NanEye viewer software (AMS, Premstaetten, Austria). The LEDStar light station (Dorc, Zuidland, The Netherlands) was used for illumination through the light fiber integrated in the PliENT endoscope.

The maxillary sinus inspection was conducted in two phases. A first phase consisted out of an inspection of both maxillary sinuses of the cadaver after a conventional medial antrostomy procedure. The second phase of the experiments consisted of the inspection of the left maxillary sinus after maxillectomies type 2, type 3 and type 4, successively. The maxillectomy type 2 was performed by extending the opening of the maxillary sinus inferiorly through removal of the medial wall together with the inferior turbinate and connecting the nasal floor with the floor of the maxillary sinus^35^. The maxillectomy type 3 was one by extending the opening anteriorly and removing the lacrimal bone^35^. For the maxillectomy type 4, the opening to the anterior wall of the maxillary sinus was extended^35^. The different enlargement procedures are illustrated in Fig. 3c. Both experimental phases are further detailed in the Method section.

#### Qualitative results

**Figure 4:**
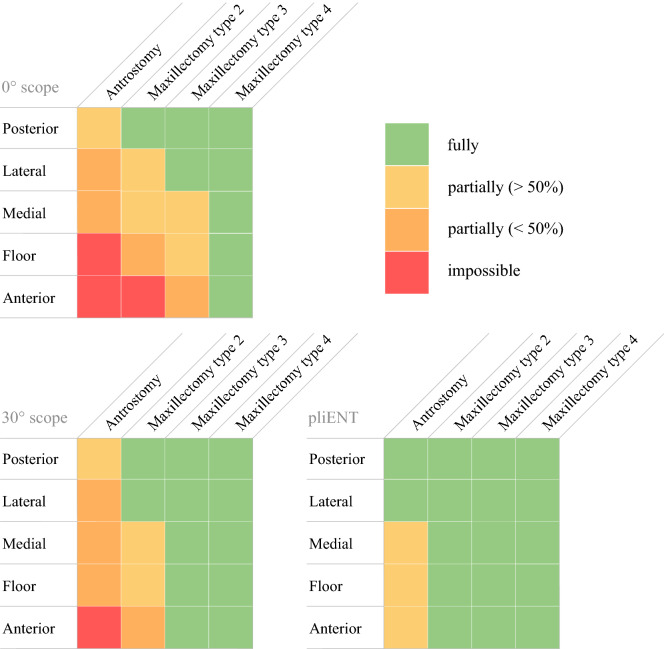
Visibility reported by the surgeons: full, partial or no visualization of the maxillary sinus walls with the 0° and 30° rigid endoscopes and the PliENT endoscope for each considered enlargement procedure.

The results regarding the surgeons’ report on the visualization of the maxillary sinus walls is presented in Fig. 4. For each enlargement procedure, the surgeons reported full, partial (more than half of the wall), partial (less than half of the wall), or no visualisation with the 0° and 30° rigid endoscopes and the pliENT endoscope. In order to calculate a mean score over the trials, each visualization grade was given a number (fully = 3; partially>50% = 2; partially > 50% = 1; impossible = 0). The mean was then rounded to the nearest integer. When a medial antrostomy was performed, the grade given for the visualization in Fig. 4 was calculated as the mean score of all trials in both maxillary sinus. When the maxillectomies were realized, the visualization grade reported in Fig. 4 was calculated as the mean score of all trials in the left maxillary sinus only. Figure 4 shows that the 0° rigid endoscope did not allow visualization of the floor (inferior wall) and the anterior walls of the maxillary sinus when a classical medial antrostomy was made. A maxillectomy type 4 was needed to visualize all maxillary sinus walls when using the 0° rigid endoscope.

The 30° rigid endoscope offered better visualization than the 0° rigid endoscope. Still, with a classical medial antrostomy, the anterior wall of the maxillary sinus could not be visualized (Fig. 4). A maxillectomy type 3 was needed to visualize all maxillary sinus walls.

The pliENT endoscope allowed to visualize all maxillary sinus walls, at least partially, when performing a classical medial antrostomy. The posterior and lateral walls could be fully inspected. The medial, inferior and anterior walls could be visualized partially at more than 50% of their full surface area. A maxillectomy type 2 was found sufficient to completely visualize all maxillary sinus walls.

In conclusion, the results reported in Fig. 4 show the added-value of the steerability property of the PliENT endoscope over the currently used 0 and 30° rigid endoscopes, i.e. visualize the maxillary sinus walls through an antrostomy while limiting the removal of additional functional structures like the lacrimal duct or the inferior turbinates. Future work will consist of testing the PliENT instrument with other existing scopes (e.g. the 70 ° endoscope) in order to generalize the added-value of PliENT to all existing scopes and not only to the most commonly used ones.

**Figure 5:**
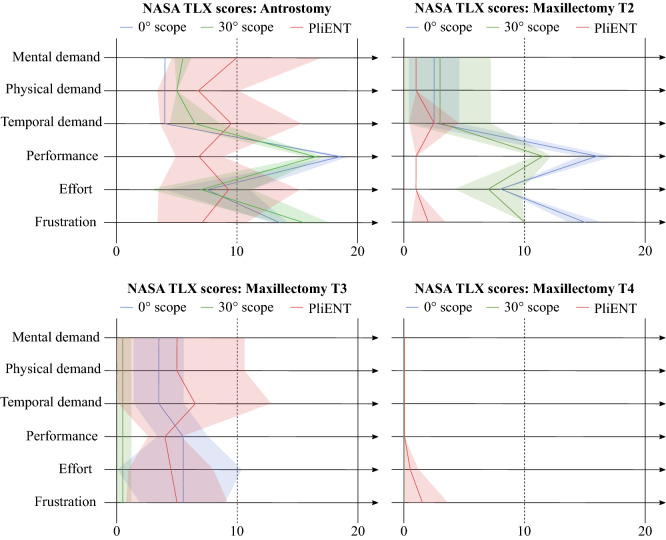
Results of the NASA-TLX test for the 0° (blue) and 30° (green) rigid endoscopes and the PliENT endoscope (red) for each of the considered enlargement procedures. The mean score is indicated with a thick line, whereas the standard deviation is indicated with a transparent surface area.

Regarding the surgeons’ experience with the different instruments, and more specifically, the workload they experienced, the NASA-TLX test indicates that handling the PliENT endoscope is both mentally and physically more demanding than operating the rigid endoscopes when only a medial antrostomy is performed (Fig. 5). However, the PliENT is believed to allow better performance and was reported to limit frustration, according to the surgeons.

This is because the surgeons could identify more walls with the flexible endoscope. The reported scores for the PliENT endoscope when a a medial antrostomy was realized show a large standard deviation (Fig. 5). This is mainly due to a difference in scoring between both surgeons. While one surgeon gave the mental, physical and temporal demand a score of 17, 5 and 19, respectively, after the first trial, the other gave a score of 2, 2 and 2, respectively. This might be due to the fact that the surgeon who gave lower scores has more experience with robotic surgery than the other surgeon. More specifically, this surgeon performs approximately 20 surgeries per year with the da Vinci surgical system (Intuitive Surgical, California, USA), whereas the other one never used such surgical robotic system before. Another possible explanation for the large standard deviation could be that there is a certain adaptation time for the surgeons to get used to this new instrument. Especially for the mental, temporal demand and the effort, a clear improvement was noticed after 6 tests. The scores improved from 17 to 11, from 19 to 7 and from 16 to 6 for the mental, temporal demand and the effort, respectively.

When a maxillectomy type 2 is performed, the PliENT endoscope scores equally or better than both rigid endoscopes for all the selected metrics (Fig. 5).

However, for maxillectomies type 3 and 4, no clear difference can be noticed in workload between instruments.

The performed NASA-TLX test assessed the PliENT endoscope as being able to enhance the surgeons’ performance and frustration. The PliENT endoscope was, however, more mentally, physically and temporally demanding than the other instruments, and asked more effort. Those observations need to be put in perspective since the PliENT endoscope was new to the surgeons and compared to instruments that each surgeons used daily. The surgeons therefore needed some trials to get used to the instrument. This learning effect is also suggested by next subsection’s analysis of the execution time of the respective inspections.

#### Quantitative results

##### Workspace

**Figure 6:**
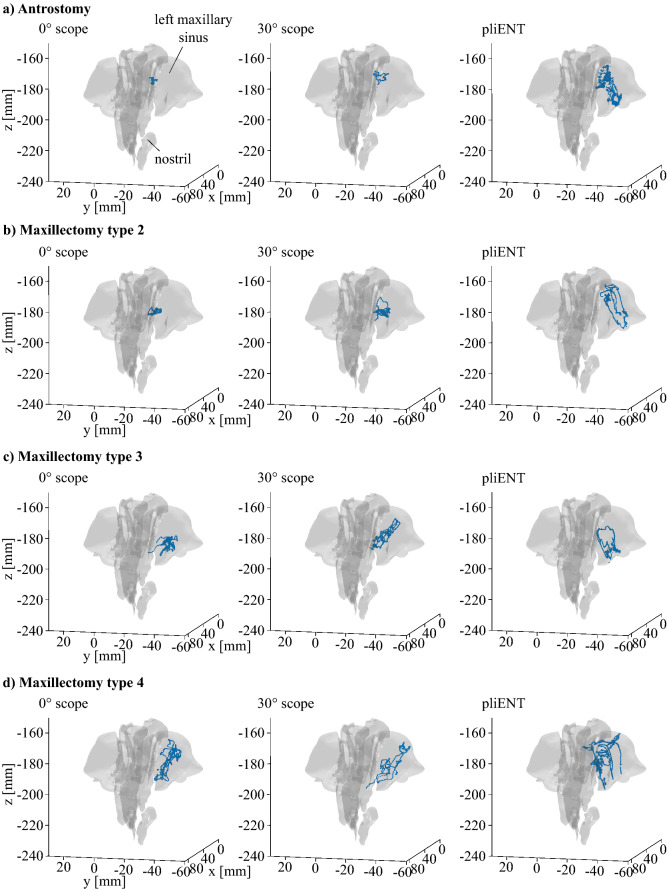
Instrument tip positions acquired by tracking of the EM sensor placed on the instruments’ tip. Tracking only takes place when the tip is inside the maxillary sinus. Each enlargement procedures is provided: (**a**) an antrotomy; (**b**) a maxillectomy type 2; (**c**) a maxillectomy type 3; (**d**) a maxillectomy type 4. Each view is a top view of the maxillary sinus and nasal cavity with the nostrils below and the posterior wall of the maxillary sinus above.

Based on the calibration detailed in the “Methods” section, the registered instrument’s tip paths can be plotted in the same frame as the 3D segmented maxillary sinus and nasal cavity. For each specific enlargement procedure, the tip position of each instrument is plotted together with the 3D segmented maxillary sinus and nasal cavity anatomy (see Fig. 6). Figure 6 gives an example of the gathered paths drawn relative to the shape of the left maxillary sinus. For each enlargement procedure and instrument, the path gathered during one test is plotted. For clarity purposes, the tip position was plotted when situated inside the maxillary sinus only. The path inside the nasal cavity was discarded as this path is similar (i.e. straight) for the three instruments. The Supplementary Figure F1 online provides a larger version of Fig. 6 for clarity purpose.

From Fig. 6, one can notice that the path made by the 0° rigid endoscope is short and compact when an antrostomy was performed. The path made by the PliENT endoscope is much larger. It reaches even up to the anterior wall. As the enlargement procedure becomes more invasive (typically for the maxillectomies types 3 and 4), the path made by the three instruments look similar in terms of volume (Fig. 6). In order to quantify this specific volume, the workspace of each instrument for each enlargement procedure was calculated using the alpha shape of the points characterizing the path, as explained in the “Methods” section. The calculated workspace volumes are reported in Table 2.

For each enlargement procedure, the largest workspace is indicated in bold in Table 2. The PliENT endoscope systematically offers a larger workspace than the rigid endoscopes and this for all the enlargement procedure. When an antrostomy is performed, the PliENT endoscope has a workspace volume that is 28 times larger than the workspace of the 0° scope. As the enlargement increases, this factor decreases. The increase in workspace goes down to a factor of only 1.8 when a maxillectomy type 4 is realized.

##### Time

The time taken by the surgeons to inspect all the maxillary sinus walls was registered. Note that the surgeons were not asked to perform the task as fast as possible, but just to observe, one by one, each maxillary sinus wall. The mean and standard deviation of the observation times are reported in Table 2. In this table, the shortest time is indicated in bold. For all the enlargement procedures, the surgeons were faster to identify the walls with the rigid instruments.

**Table 2:** Mean and standard deviation of the time taken for inspection of all the walls of one maxillary sinus, the workspace corresponding to the volume of the alpha shape calculated from the position data of the EM sensor placed at the instrument’s tip and bending angle of the PliENT endoscope.

Metric	Instrument	Antrostomy	Maxil. T2	Maxil. T3	Maxil. T4	Total
Workspace [mm^3^]	0° scope	12.6 ($\pm 14$)	47.8	294.2	391.6	186.6 ($\pm 185.4$)
	30° scope	54.5 ($\pm 31.4$)	140.4 ($\pm 38$)	139.1 ($\pm 1$)	228.6 ($\pm 45.7$)	140.6 ($\pm 71$)
	PliENT	**348.9** ($\pm 275.7$)	**323** ($\pm 143.7$)	**762.9** ($\pm 145.4$)	**706.3**	**535.3** ($\pm 231.5$)
Time [s]	0° scope	**55** ($\pm 7$)	**50** ($\pm 8.5$)	73 ($\pm 21.2$)	52.5 ($\pm 21.9$)	**57.6** ($\pm 15.6$)
	30° scope	55.5 ($\pm 6.4$)	73 ($\pm 40$)	**65** ($\pm 11.31$)	**45.5** ($\pm 6.4$)	59.8 ($\pm 19.4$)
	PliENT	114.6 ($\pm 34.5$)	83 ($\pm 10$)	114 ($\pm 1.41$)	90 ($\pm 56.57$)	102.8 ($\pm 30$)
Bending angle: $\int {\delta (\alpha )}$ [°]	PliENT	6.4 ($\pm 2.3$)	5.3 ($\pm 0.3$)	7.2 ($\pm 2.8$)	6.2 ($\pm 5.7$)	–

Each metric was measured/calculated for each instrument and for each considered enlargement procedure. The shortest time and the largest workspace is indicated in bold for each enlargement procedure. The last column (marked **Total**) lists mean and standard deviation of all the tests realized with a specific instrument, accumulated over all the enlargement procedures.

There are four potential explanations for this:

First, the flexible instrument was new to them. This means that the surgeons took some time to get used to the instrument, especially at the beginning of the experiment, when they needed to test the instrument with an antrostomy as enlargement procedure. However, the time decreased with the number of trials, which indicates that the surgeon could identify faster all the maxillary sinus walls as the number of trials increased. This could explain the large standard deviation reported in Table 2. The fact that the surgeon needed more time at the beginning of the experiments to get used to the instrument is also visible in the results of the NASA-TLX test. The surgeons indeed reported that using the PliENT endoscope was more temporally demanding when a medial antrostomy was realized.

Second, as reported in Fig. 4, using the 0° and 30° rigid endoscopes did not allow the visualization of all the walls, especially when an antrostomy, a maxillectomy type 2 and type 3 was realized. In those cases the task stopped prematurely, which also explains why the time for inspection was shorter for both rigid endoscopes when an antrostomy, a maxillectomy type 2 and type 3 were performed.

Third, the interface of the PliENT endoscope is more complex as there is an extra actuation button to operate compared to rigid endoscopes. The surgeons needs to take some time to actuate this extra DOF. Where this was found mentally, physically and temporally demanding when an antrostomy was performed (Fig. 5), the surgeons could get rapidly used to it as the trials increased and the enlargement procedure was more invasive (Fig. 5). Note that this decrease in workload as the enlargement increases is not due to the fact that the surgeon stopped using the flexible DOF. Surgeons did not use the flexible endoscope similar as a rigid one and kept exploiting the extra DOF that was available. This reasoning is further detailed in the following subsection.

Finally, the camera used in the PliENT endoscope is a low-cost chip-on-tip camera which provides lower image quality than the rigid endoscopes. This made the inspection and the identification of the maxillary sinus walls more challenging with the PliENT endoscope. This forms another explanation for the longer duration. The Supplementary Video V1 online shows some endoscopic views from the PliENT endoscope.

##### Bending angle

The bending angle of the PliENT endoscope was recorded by tracking both EM sensors fixed on the instrument. One was placed on the tip (bending) as the other was placed on the shaft (fixed) (Fig. 3a). The difference between their orientations was then calculated. A metric was defined to compare how often the bending DOF of the PliENT endoscope was used by the surgeons. This metric was defined as the integral of the absolute value of the derivative of the bending angle over the task time. The value of this metric is reported in Table 2.

No large difference in terms of activation of the bending DOF of the flexible instrument was seen between the different enlargement procedures. This means that the surgeons used the flexibility of the PliENT endoscope in a comparable manner when the anatomical workspace was highly constrained (when only an antrostomy was realized) as when the anatomical workspace was less constrained (when a maxillectomy type 4 was realized). The decreasing workload that was felt by the surgeons using the PliENT endoscope as the enlargement became larger (Fig. 5) is therefore expected not to be due to the fact that they refrained from using the bending angle and hence rather used the flexible endoscope as a rigid scope.

##### Field of view through a medial antrostomy

**Figure 7:**
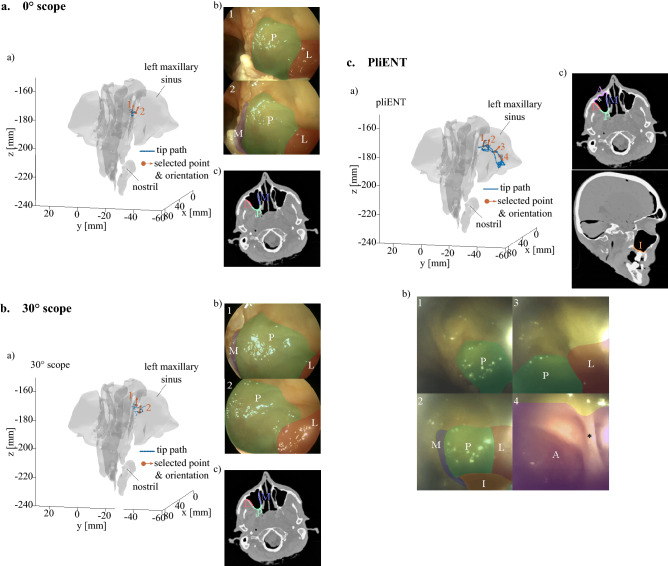
Field of view from points (points 1, 2,3 and 4) taken from the path made by the (**a**) 0° scope, (**b**) 30° scope, and (**c**) PliENT scope during inspection of the left maxillary sinus through an antrostomy. (a) 3D path (blue points) and selected points (orange points). The orientation of the instrument at the selected points is indicated by an arrow; (b) Endoscopic view at the selected points with highlight of the different maxillary sinus walls; (c) CT scan in the transverse plane of the cadaver head with highlight of the different walls visible on the endoscopic views. P = posterior wall; L = lateral wall; M = medial wall; A = anterior wall; I = inferior wall or floor; (*) partial septum.

In order to check whether the increase in workspace when using the PliENT endoscope also leads to an increased view upon the anatomy, the recorded camera images were analyzed for the three instruments (Fig. 7). As illustration, one inspection task of the left maxillary sinus walls was chosen for each instrument when an antrostomy was performed (Fig. 7).

Figure 7a shows that only the posterior, lateral and medial wall could be seen partially with the 0° scope. The inferior part of the posterior wall is indeed not visible on the endoscopic views, whereas only a small part of the lateral and medial wall can be seen. Those endoscopic images of the limited field of view of the 0° scope support the surgeon’s report during maxillary sinus inspection through an antrostomy (Fig. 4).

Figure 7b shows that only partial views upon the posterior, lateral and medial wall are possible with the 30° scope. This observation confirms the surgeon’s comments reporting only partial views of the posterior, lateral and medial wall.

Figure 7c shows that the PliENT endoscope can at least visualize all the maxillary sinus walls partially. Also this was reported by the surgeons (Fig. 4). More specifically, the posterior and lateral walls could be visualized completely and the anterior, medial and inferior walls could be seen partially. A partial septum visible on the cadaver’s head CT scan (indicated with the star symbol (*) in Fig. 7c(c)) is also clearly visible on the endoscopic view (Fig. 7c(b), view from point 4). This feature is situated on the anterior wall, which proves that the PliENT endoscope can partially visualize the anterior wall. The maximum bending angle of PliENT (93°) limits the full visualization of the medial, floor and anterior wall. Therefore, future work will focus on increasing the bending angle of the instrument. This can be realized by using a longer McKibben muscle inside the handle of the instrument, or using a dynamic join inside the McKibben muscle, between both tubes in order to reduce friction.

Figure 7c(b) shows the somewhat poorer image quality provided by the PliENT endoscope compared to the image quality of the rigid endoscopes (Fig. 7a(b) and b(b)). The surgeons reported an increased difficulty in identification of the maxillary sinus walls because of the poorer image quality. This comes from the use of a low-cost chip-on-tip camera in the endoscope (i.e. the NanEye camera with a resolution of 249 × 250 pixels). This camera was chosen for its low cost and easy integration and use considering that the PliENT instrument is a first prototype. The NanEye camera was good enough to provide a first proof of concept, however, it should be replaced by higher resolution one at a further development stage. For example, the OVM6946 400 × 400 camera (OmniVision, California, USA) which as approximately the same footprint than the NanEye camera could be considered. Note that we are dependent on camera technologies, which are expected to improve in the future towards even higher resolution for a very low footprint.

## Methods

### Instrument assembly

The PliENT endoscope for maxillary sinus surgery is intended to be disposable. It has a cylindrical handle of 22 mm outer diameter. The handle is 3D printed using a Vero white RGD835 material and is made of two parts (parts 5 in Fig. 2c). Both parts of the handle fit together one upon another and are fixed using a plug-on cap proximally and a screw-on cap distally (components 10 and 4 in Fig. 2c, respectively).

On the upper portion of the handle, two push buttons are embedded for commanding the embedded actuators that steer the flexible distal tip. The buttons are obtained by cutting and modifying an Adafruit keypad (Adafruit Industries, New York, USA) from a 1 × 4 towards a 1 × 2 formfactor. As such, it fits the user interface frame integrated in the handle (part 6 in Fig. 2c). In order to make the endoscope actuation intuitive for the surgeons, the interface was placed in line with the bending plane of the distal part. The surgeon therefore knows that the bending direction is aligned with the longitudinal symmetry plane of the keypad.

Inside the handle, a 6 mm diameter McKibben pneumatic artificial muscle with integrated channel, introduced by our group in earlier work^36^, is used as linear actuator for bending the distal tip (part 9 in Fig. 2c). A McKibben artificial muscle is a linear actuator operated by pressurized air. It is composed of an internal bladder reinforced by a non-extensible braided mesh. The internal bladder is connected to an air supply^37^. By increasing the air pressure, the bladder tends to expand radially. Since the braid length is constant, the braid angle increases and the actuator contracts. The Supplementary Video V1 online shows the basic principle of a McKibben artificial muscle. The McKibben artificial muscle is here chosen as actuator because of its miniaturization potential, large power density and operational bandwidth and compliant behaviour, which offers safety to the patient^37^. The muscle is slid onto and fixed at one end to a mobile stainless-steel outer tube with an external diameter of 1.8 mm and a wall thickness of 0.1 mm, and at its other end to fixed stainless-steel inner tube of 1.5 mm external diameter and 0.1 mm wall thickness (parts 7 and 8 in Fig. 2c). This system of two tubes surrounded by a muscle was introduced under the name: concentric muscle^36^. The fixed inner tube acts as a working channel where the camera and light source can be inserted. In order to ensure the muscle fixation, a conical ring is slid onto the muscle at the level of the attachment inside the handle. The whole muscle fixation is secured by adding a small quantity of epoxy glue (Loctite EA 3430, Düsseldorf, Germany).

In the handle, at the level of the muscle attachment, a duct has been created that serves as a connection to an air supply that drives the muscle. Proximally, a thin tube ensures the connection between the fixed handle-muscle attachment piece and the pressure connector (component 12 in Fig. 2c). The pressure connector, a QSM mini Festo push-in connector (Festo, Esslingen am Neckar, Germany, component 11 in Fig. 2c) ensures easy plug-in of an external pressure source.

Distally, a stainless-steel steer cable of 0.2 mm diameter (Carl Stahl, Süßen, Germany) is welded using silver to the distal part of the mobile outer tube of the McKibben muscle (part 3 in Fig. 2c). This cable is glued to the distal tip using epoxy glue 204-CTH (Dymax Corp, Torrington, USA). The tip is glued as well to the distal end of the NiTi notched tube (2.3 mm inner diameter, 0.15 mm wall thickness and 200 mm long). Latter tube covers the tubes of the concentric McKibben muscle. The NiTi shaft has been heat-treated prior to manufacturing. More specifically, the sample was annealed at 550 °C for 10 mins. This heat treatment allows to decrease the NiTi stress plateau and therefore the load required to bend the NiTi backbone^38^. The notches were cut into the NiTi shaft using the wire-EDM manufacturing process. The shaft is fixed to the handle using a clamp ring intrinsically printed inside the handle (part 5 in Fig. 2c). The tip of the endoscope is 3D printed using the HTM 140 material. Finally, a NanEye camera (AMS, Premstaetten, Austria) and a light fiber (Dorc, Zuidland, The Netherlands) are inserted inside the distal tip and glued using epoxy (Fig. 2b). The NanEye camera is a relatively cheap chip-on tip camera that was already used in previous studies on surgical instrumentation^39,40^.

The whole endoscope is 370 mm long and only weights 51 g. Its shaft has a diameter of 2.3 mm and the handle, a diameter of 22 mm. The PliENT endoscope is, to our knowledge, the most slender, active, steerable endoscope ever made for maxillary sinus surgery (Fig. 2a). In order to avoid the insertion of a sensor in the distal tip, which would increase the instrument diameter, a feedforward control scheme was adopted in order to control the distal tip. More specifically, a modified generalized Prandtl–Ishlinski model described in our previous publication was used^41^. The instrument can bend up to 93° by pushing on the upper button of the interface. The lower button on the interface allows to decrease the instrument bending angle. The maximum bending rate was set to approximately 11° per second. This bending rate is measured when producing a continuous push on the interface button. The system was set to allow more precise control with a short push. Upon a short button push, the instrument would bend 0.3°. This was done to allow small and precise adjustments upon wish. This specific slow rate was chosen for the experiments in order to avoid sudden changes in bending, and allow the surgeon to precisely inspect the maxillary sinus cavity. Note that the bending rate of the instrument can be adapted in order to match the surgeon’s preference.

Even if the instrument is intended to be disposable, it does not suppress the need for initial sterilization. Similar to other passive flexible endoscope low-temperature sterilization approaches like Vaporized Hydrogen Peroxide (H2O2) or Vaporized Ethylene Oxide (EtO) sterilization would be the most recommended ways to sterilize this instrument^42^.

### Experimental phases

The PliENT instrument was tested by two surgeons. The first surgeon has 9 years experience with surgery but no experience with surgical robotic systems whereas the second surgeon has 22 years experience with surgery and performs on average 20 procedures per year on the da Vinci surgical system (Intuitive Surgical, California, USA). Both surgeons guided the design of the PliENT instrument since the beginning by helping identifying the instrument’s specifications. The instrument design was presented and demonstrated to the surgeons the day before the experiments. On the day of the experiments, two experimental phases were conducted.

#### First experimental phase

One of the ENT surgeon first performed a classical medial antrostomy (i.e. the least invasive of the considered enlargement procedures) in both maxillary sinuses of the cadaver. When the procedure was completed, a 6 DOF electromagnetic sensor was used to scan the forehead and the nose of the cadaver’s head. More specifically, the 6 DOF sensor was placed perpendicularly to the cadaver skin with the tip touching the skin and was moved on the cadaver’s forehead and nose in order to obtain a 3D surface of the forehead and the nose. This 3D surface is used in a later phase for the calibration of the instrument tip with respect to the cadaver’s maxillary sinus.

After scanning the cadaver’s head for calibration, the PliENT endoscope was given to two ENT surgeons for 10 mins to get acquainted with its bending capabilities. This took place outside of the cadaver such that surgeons could appreciate better the behaviour of the flexible scope. Both surgeons were then asked to systematically inspect the posterior, lateral, anterior, inferior and medial wall of the maxillary sinus (following this specific order) using, first, the 0° rigid endoscope, second, the 30° rigid endoscope, and finally, the PliENT endoscope. Those tasks were first realized inside the left maxillary sinus and then conducted in the right maxillary sinus.

During inspection of the maxillary sinus walls, the surgeons were asked to indicate whether they obtained a full, partial or no visualization of each wall. These results were noted down in a logbook. The instrument’s tip positions (and bending angle for the PliENT endoscope) and the endoscopic views were recorded all along the experiments. For each instrument, the time it took to inspect all the walls of one sinus was noted down as well.

After each full maxillary sinus inspection and for each instrument, the surgeons were asked to fill in a NASA-TLX (Task Load Index) test^43^. This test is commonly used to assess the perceived workload. Here, it helps comparing the surgeon’s workload across each instrument^44–46^. In these experiments, the surgeons were asked to indicate on a 20-point scale how mentally and physically demanding the task (i.e. an inspection of all the maxillary sinus walls in one specific maxillary sinus using one specific instrument) was, how hurried or rushed the pace of the task was, how successful the surgeons were in accomplishing what they were asked to do, how hard did they have to work to accomplish their level of performance, and how insecure, discouraged, irritated, stressed, and annoyed the surgeons were during the task. This NASA-TLX test hence provides a subjective qualitative assessment of the PliENT endoscope compared to both rigid endoscopes.

The 0° and 30° rigid endoscopes were tested once in each maxillary sinus, whereas the PliENT endoscope was tested three times in each sinus. The PliENT endoscope was operated repetitively so that we could assess whether surgeon’s needed some adaptation time when using such new instrument.

#### Second experimental phase

After completion of the first phase, the surgeons realized a maxillectomy type 2 in the left maxillary sinus only (Fig. 3c). Again, systematic inspection and report of full, partial or no visualization of the maxillary sinus walls was asked to both surgeons using, first, the 0° rigid endoscope, second, the 30° rigid endoscope, and finally, the PliENT endoscope. The same metrics as mentioned in the first experimental phase were recorded in this second phase. The NASA-TLX test was also completed by the surgeons after each inspection with each endoscope. Each endoscope was tested once.

This experimental scheme was repeated after realization of a maxillectomy type 3 and type 4 (i.e. the most invasive of the considered enlargement procedures).

### Calibration

**Figure 8:**
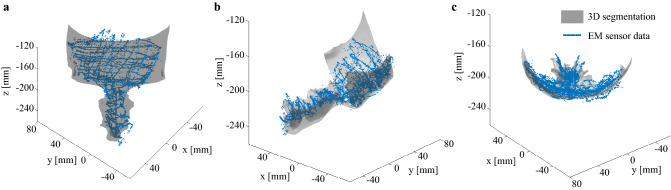
Matching of the 3D segmentation obtained from the cadaver’s head CT scan and the data obtained from manual scanning of the cadaver’s head forehead and nose skin using an EM sensor using the *pcregistericp()* function of Matlab. (**a**) frontal view; (**b**) lateral view; (**c**) top view.

In order to analyze the instruments’ tip path during inspection of the maxillary sinus, the data obtained by manual scanning of the forehead and the nose using an EM sensor were matched with the 3D segmentation of the forehead and nose skin of the cadaver. Those specific anatomical sites were chosen because of their relative closeness to the maxillary sinus and because the skin on those sites is supported by hard tissues (bone and cartilage), which makes the manual scanning of the skin with an EM sensor on those sites easier and more accurate as if the skin was supported by soft tissues. The segmentation was obtained after acquisition of a CT scan of the cadaver’s head before the experiments. The CT scan was acquired using a SOMATOM Force CT Scanner (Siemens Healthineers, Erlangen, Germany) with a 0.46 mm × 0.46 mm pixel resolution in plane and a slice thickness of 0.4mm. The CT scan was manually segmented using Mimics (version 20.0, Materialise, Leuven, Belgium). The 3D segmentation of the forehead and the nose was converted in a point cloud using the software 3-matic research (version 12.0, Materialise NV, Leuven, Belgium). The transformation matrix between the point cloud and the data acquired from the EM sensor was found using the *pcregistericp()* function of Matlab (MathWorks, Massachusetts, USA), which register two point clouds using an ICP algorithm. The resulting matched EM sensor data to the 3D segmentation is plotted in Fig. 8.

### Workspace calculation

In order to calculate the workspace of each instrument inside the maxillary sinus, the alpha shape of the points characterizing the path was calculated using the Matlab’s function *alphaShape()*. An alpha shape is a bounded volume that envelops a set of 3D points^47^. This function makes use of a generalized disk of radius 1/$\alpha _s$ for each of the 3D points.

An edge of the alpha shape is drawn between two points if a generalized disk of radius 1/$\alpha _s$ does not contain any point of the point set and if those two points lie on the boundary of the generalized disk^48^. To calculate the workspace of each instrument, the default value of $\alpha _s$ was chosen, which is the smallest $\alpha _s$ radius that produces an alpha shape enclosing all the points (Supplementary Video).

The volume of those alpha shapes was then calculated using the Matlab’s function *volume()*. The calculated workspaces are reported in Table 2. In the case of the antrostomy, 8 tests were conducted in total with the PliENT instrument (4 tests inside each maxillary sinus, in total, 6 tests were performed by one surgeon, and 2 tests by the other) and 2 tests (1 test in each sinus performed by one surgeon) were conducted with the 0° and 30° rigid endoscopes. For the other enlargement procedures, two tests were realized (one test per surgeon) on the left maxillary sinus using each instrument. A mean and a standard deviation could then be calculated for each instrument and each enlargement procedure. In Table 2, however, some entries do not mention any standard deviation. This is due to the fact that for some tests, the data acquired by the EM sensor was not usable (because of failure of tracking for a couple of seconds) leaving only one test for the considered instrument and enlargement procedure.

### Informed consent

The donor or next of kin provided informed consent.

## Supplementary Information

Supplementary Legends.

Supplementary Figures.

Supplementary Video S1.

## Data Availability

The main data supporting the results of this study are available within the paper. The unprocessed data are available from the corresponding author upon request.

## Code availability

The segmentation of the nasal cavities and maxillary sinuses were performed using Mimics (version 20.0, Materialise, Leuven,Belgium). The segmentations were converted in a point cloud using the software 3-maticresearch (version 12.0, Materialise, Leuven, Belgium). The data processing was realized using Matlab R2017a (MathWorks, Massachusetts, USA).
