# Supplementary material for: A miniature robotic steerable endoscope for maxillary sinus surgery called PliENT
Source: Sci Rep. 2022 Feb 10;12:2299. doi: 10.1038/s41598-022-05969-3 (PMC8831515; doi:10.1038/s41598-022-05969-3)
Supplement: Supplementary file 1 — Supplementary Legends. [file 41598_2022_5969_MOESM1_ESM.docx]

Supplementary Video V1: Video of the PliENT instrument showing bending capabilities, actuation principle and endoscopic views. Note that the instrument does not exactly return to 0° when the instrument relaxes. Instead, the rest bending position of the instrument is 15°. However, since the instrument is very small (2.3mm diameter), the surgeon can incline it inside the maxillary sinus to compensate for this offset present in the rest bending position.
