# Supplementary material for: A miniature robotic steerable endoscope for maxillary sinus surgery called PliENT
Source: Sci Rep. 2022 Feb 10;12:2299. doi: 10.1038/s41598-022-05969-3 (PMC8831515; doi:10.1038/s41598-022-05969-3)
Supplement: Supplementary file 2 — Supplementary Figures. [file 41598_2022_5969_MOESM2_ESM.pdf]

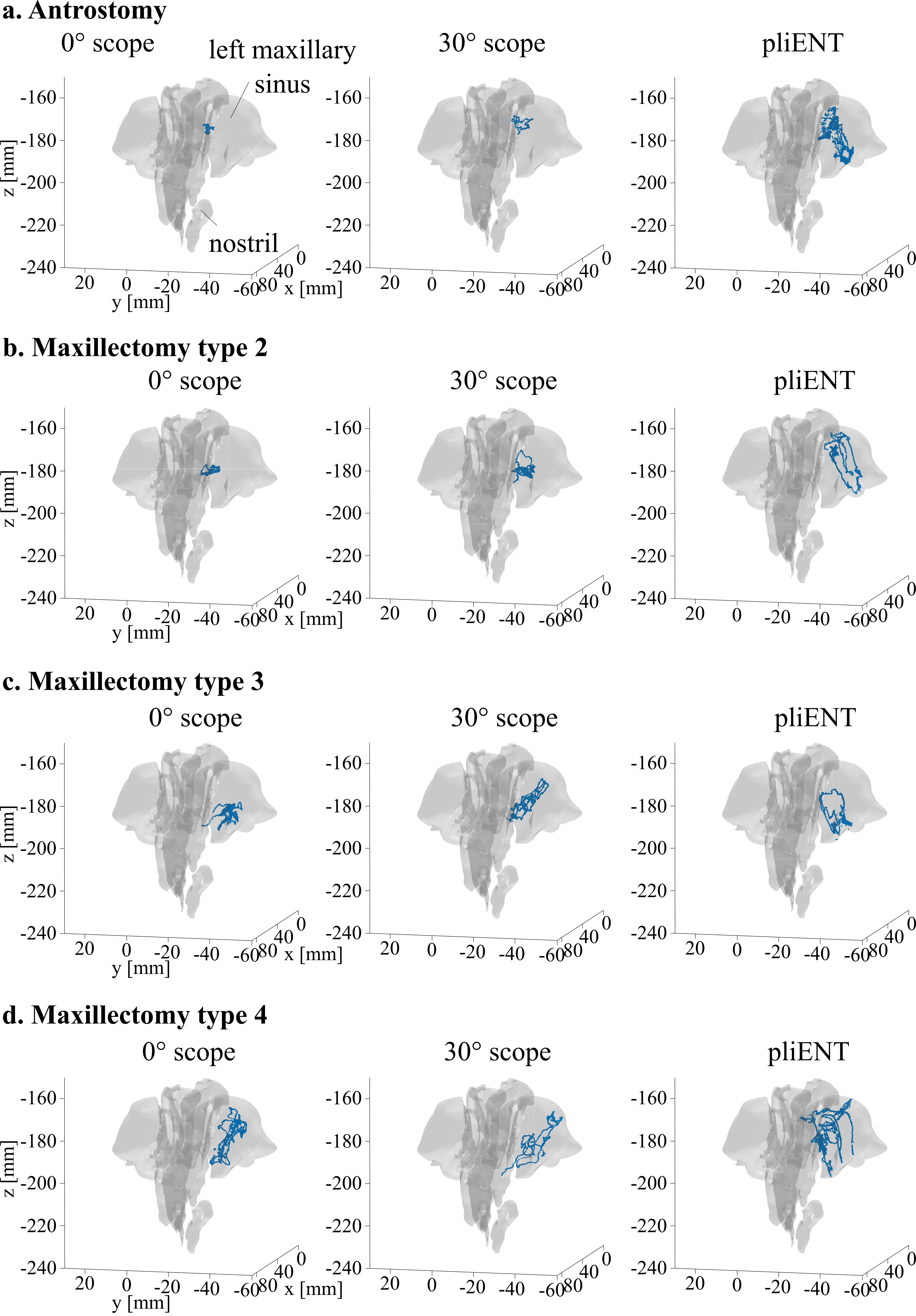

Supplementary figure F1:

Instrument tip positions acquired by tracking of the EM sensor placed on the instruments' tip. Tracking only takes place when the tip is inside the maxillary sinus. Each enlargement procedures is provided: a) an antrotomy; b) a maxillectomy type 2; c) a maxillectomy type 3; d) a maxillectomy type 4. Each view is a top view of the maxillary sinus and nasal cavity with the nostrils below and the posterior wall of the maxillary sinus above.
